# Proapoptotic effect of endocannabinoids in prostate cancer cells

**DOI:** 10.3892/or.2015.3746

**Published:** 2015-01-21

**Authors:** O. ORELLANA-SERRADELL, C.E. POBLETE, C. SANCHEZ, E.A. CASTELLÓN, I. GALLEGOS, C. HUIDOBRO, M.N. LLANOS, H.R. CONTRERAS

**Affiliations:** 1 Physiology and Biophysics Program, Institute of Biomedical Sciences, Faculty of Medicine, University of Chile, Santiago 8389100, Chile; 2 Pathological Anatomy Service, Clinic Hospital of the University of Chile, University of Chile, Santiago 8389100, Chile; 3 Urology Service, Clinic Hospital of the University of Chile, University of Chile, Santiago 8389100, Chile; 4 Laboratory of Nutrition and Metabolic Regulation, INTA, University of Chile, Santiago 8389100, Chile

**Keywords:** prostate cancer, endocannabinoid receptors

## Abstract

In the early stages, prostate cancer is androgen- dependent; therefore, medical castration has shown significant results during the initial stages of this pathology. Despite this early effect, advanced prostate cancer is resilient to such treatment. Recent evidence shows that derivatives of *Cannabis sativa* and its analogs may exert a protective effect against different types of oncologic pathologies. The purpose of the present study was to detect the presence of cannabinoid receptors (CB1 and CB2) on cancer cells with a prostatic origin and to evaluate the effect of the *in vitro* use of synthetic analogs. In order to do this, we used a commercial cell line and primary cultures derived from prostate cancer and benign prostatic hyperplasia. The presence of the CB1 and CB2 receptors was determined by immunohistochemistry where we showed a higher expression of these receptors in later stages of the disease (samples with a high Gleason score). Later, treatments were conducted using anandamide, 2-arachidonoyl glycerol and a synthetic analog of anandamide, methanandamide. Using the MTT assay, we proved that the treatments produced a cell growth inhibitory effect on all the different prostate cancer cultures. This effect was demonstrated to be dose-dependent. The use of a specific CB1 receptor blocker (SR141716) confirmed that this effect was produced primarily from the activation of the CB1 receptor. In order to understand the MTT assay results, we determined cell cycle distribution by flow cytometry, which showed no variation at the different cell cycle stages in all the cultures after treatment. Treatment with endocannabinoids resulted in an increase in the percentage of apoptotic cells as determined by Annexin V assays and caused an increase in the levels of activated caspase-3 and a reduction in the levels of Bcl-2 confirming that the reduction in cell viability noted in the MTT assay was caused by the activation of the apoptotic pathway. Finally, we observed that endocannabinoid treatment activated the Erk pathway and at the same time, produced a decrease in the activation levels of the Akt pathway. Based on these results, we suggest that endocannabinoids may be a beneficial option for the treatment of prostate cancer that has become nonresponsive to common therapies.

## Introduction

Prostate cancer (PrC) is a highly prevalent oncologic pathology in most countries throughout the world (1). During its early stages this disease is usually asymptomatic and exhibits slow progression, which carries the risk of having it diagnosed at an advanced stage. Research indicates a direct correlation between the appearance of symptoms and the spreading of the cancer or metastasis. This late diagnosis may decrease the treatment options available for patients and also the chances of recovery (2).

Due to the above factors and in light of the aggressive nature of this disease in its late stages, it has become critical to search for more effective tools with which to detect PrC while it is still in its early stages, and for better treatment options. These two aspects would greatly improve the quality of life and the overall survival expectancy of these patients (3,4).

Regarding the molecular pathogenesis of PrC, it has been observed that certain genetic alterations may provoke the transformation of normal prostatic cells into cancerous cells. In this context, it has been reported that certain mutations in key specific genes such as PTEN, TP53, E-cadherin and β-catenin (5) are important in this transformation process. In addition, it has been demonstrated that several growth factors, such as insulin growth factor I (IGF-I), transforming growth factor α and β (TGFα or β) and members of the fibroblast growth factor (FGF) family, may be involved in the proliferation and metastasis of PrC (6).

The molecular factors involved in the development of PrC are various and as the transformation process in the cell evolves, it acquires a malignant phenotype with the ability to invade and generate metastasis in different parts of the body, but with a preference to form metastatic lesions in the bones (5,6). For this reason, many compounds have been used to try and control the progression of prostate cancer. It has been suggested that marijuana (*Cannabis sativa*) through some of its active compounds could be involved in halting tumoral growth and thus delaying its progression to more advanced and aggressive stages (7–9).

Marijuana acts in the organism as a psychoactive agent through the production of its active component, the cannabinoid (−)-Δ^9^-tetrahidrocanabinol (or THC), which is an aromatic terpenoid compound with a very low solubility in water. Two different cannabinoid receptors have been described, characterized and cloned: cannabinoid receptor 1 (or CB1), originally found in the brain, and cannabinoid receptor 2 (or CB2), which was first described in the spleen. Both receptors are part of the superfamily of G protein-coupled receptors (10,11).

Other than its exogenous ligands such as THC, the organism can produce similar compounds that have been termed as endocannabinoids, which are capable of modulating several physiological mechanisms through the CB1 and CB2 membrane receptors (12), although it has also been reported recently that they may also act in an independent receptor manner (13).

Endocannabinoids are molecules that are derived from unsaturated fatty acids acting as endogenous ligands for cannabinoid receptors CB1 and CB2 and originate in the plasma membrane from phospholipids in response to a rise in the intracellular concentrations of calcium. These endogenous ligands bind to the CB1 and CB2 receptors with great affinity and participate in many biological processes in the immune, respiratory, circulatory and reproductive systems (9,14–17). Endocannabinoids are then eliminated in a two step process: first, an intracellular accumulation, followed by an enzymatic metabolization by an enzyme that belongs to the serine hydrolase family, the fatty acid amide hydrolase (or FAAH) or by the monoacyl glycerol lipase (a soluble serine hydrolase enzyme). These two enzymes are the main proteins in charge of degrading endocannabinoids (18).

On a cellular level, it has been found that endocannabinoids may modulate cell proliferation, viability and differentiation. This evidence suggests that endocannabinoids may also be involved in controlling the growth and transformation of tumor cells (8,19,20). Regarding this, evidence shows that endocannabinoids may inhibit the growth of several types of tumors through the inhibition of proliferative pathways such as adenylate cyclase (21) and protein kinase A (22), arrest of the cell cycle by induction of p27 (23), downregulation of the EGF receptor (EGF-R) and other molecules related to growth pathways such as the nerve growth factor receptor (NGF-R), the vascular endothelial growth factor (VEGF) and prolactine (24). Likewise, when tumor cells are treated with specific antagonists for endocannabinoids, the invasive ability of tumors increases. The great advantage that the use of endocannabinoids may bring to the battle against prostate cancer is that it has been demonstrated that the receptors for these molecules are substantially overexpressed in cancerous prostatic cells when compared to normal, healthy prostate tissues (9,25).

There are two classes of endocannabinoids. Among those that are derived from fatty acids, anandamide is the most studied and was the first to be described. Other members of this family include N-oleoylethanolamide and N-palmitoylethanolamide, which have shown strong dietetic effects independent of CB1 or CB2 activation. The second class of endocannabinoids are those bound to glycerol, of which 2-arachidonoyl glycerol (2-AG) is the most commonly used in research due to its demonstrated effects on cancer cells. Another member of this family is 2-arachidonyl glyceryl ether, the endogenous relevance of which is currently being studied (9).

Due to the previous effects noted in other types of cancer and increased knowledge of anandamide (and its synthetic analog methanandamide) and 2-AG, further investigation of the effects of these endocannabinoids for the treatment of PrC is warranted. Recently, it was reported that an increment in intracellular levels of 2-AG inhibited the invasive ability of the PrC cell lines PC3 and DU-145 by a mechanism involving the activation of the CB1 receptor and through the inactivation of protein kinase A (22). In the same manner, the direct participation of anandamide (Ana) in decreasing the proliferative action of EGF in cell lines has been proven. Following treatment with anandamide in DU-145 and LNCaP cells, a downregulating effect on the expression levels for the receptor of the EGF growth factor accompanied by a proliferative arrest in the G1 phase of the cell cycle and a rise in the levels of apoptosis and necrosis in the cells were found (26–28).

Not with standing these results, information concerning the signaling pathways mediating these effects in PrC is still scarce and somewhat contradictory in nature. Some information has been reported in other types of cancer, for example, the activation of the AKT pathway in astrocytoma cells after treatment with THC or Ana (29), activation of the ERK pathway when using THC in glioma cell lines (30) or the activation of the JNK pathway when using endocannabinoids in different types of nerve cells that express the CB1 receptor (31).

To date, all of the studies using endocannabinoids have been made in cell lines. The objective of the present study was to analyze the effect of endocannabinoids, not only on cell lines, but also on primary cultures of PrC and the signaling pathways involved in order to obtain a better understanding of the possible effects following treatment with these molecules against prostate cancer.

## Materials and methods

### Materials

Endocanabinoids anandamide (Ana) and 2-AG were purchased from Calbiochem (San Diego, CA, USA) (cat. nos. 172100 and 181251, respectively) and methanandamide (Me) was purchased from Biomol (Plymouth Meeting, PA, USA) (cat. no. FA-021). CB1 receptor antagonist, SR141716, was purchased from Sanofi (Montpellier, France).

Primary antibodies for the CB1 and CB2 receptors were obtained from Cayman Chemical (Ann Arbor, MI, USA) (cat. nos. 10006590 and 101550, respectively), and caspase-3 and Bcl-2 from Cell Signaling Technology, Inc. (Danvers, MA, USA) (cat. nos. 9661 and 2876, respectively). Phospho-p44/42 MAPK (Erk1/2) was purchased from Cell Signaling Technology, Inc. (Danvers) (cat. no. 9101) and p-Akt 1/2/3 (Thr308)-R from Santa Cruz Biotechnology, Inc. (Dallas, TX, USA) (cat. no. 16646-R). Anti-mouse and anti-rabbit secondary peroxidase-conjugated antibodies were obtained from Jackson Immuno Research (West Grove, PA, USA) (cat. nos. 115-035-003 and 111-035-003, respectively). Anti-rabbit FITC-conjugated secondary antibodies were obtained from Jackson Immuno Research (cat. no. 305-095-003). For immunohistochemistry the subsequent kits were used: Histostain^®^-Plus Bulk kit (cat. no. 85-8943) and the DAB-Plus Substrate kit (cat. no. 00-2020) (both from Invitrogen, Carlsbad, CA, USA). Annexin V-FITC Apoptosis Detection kit was obtained from BD Pharmingen (Franklin Lakes, NJ, USA) (cat. no. 556547).

### Methods

#### Immunohistochemistry

Formalin-fixed and paraffin-embedded prostate specimens were obtained from the archives of the Pathological Anatomy Service, Clinic Hospital of the University of Chile, with the corresponding authorization. All samples were evaluated by an expert pathologist (I.G.) and grouped as following: benign prostate hyperplasia (BPH) as a non-malignant control and PrC samples of high and low Gleason score. Tumor and control samples were cut into 5-μm sections, mounted on silane-treated slides, deparaffinized in xylene and dehydrated in a series of ethanol solutions with increasing ethanol content up to 100%. The sections were washed with phosphate-buffered solution (PBS) (5 min, 3 times). The sections were incubated in a steam bath for 10 min at 95–100°C in retrieval buffer (10 mM citrate buffer, pH 6.0). After cooling down, the samples were incubated with 3% H_2_O_2_ for 10 min, in order to inhibit the activity of endogenous peroxidase. Then, the sections were washed with PBS 2–3 times, incubated in blocking solution (PBS 2% BSA) at room temperature for 1 h and washed again with PBS 2–3 times. Primary antibodies were added and the sections were incubated for 1 h at 37°C or overnight at 4°C. After incubation with the primary antibodies, a secondary antibody was added to the sections for 30 min at 37°C. Then, the samples were washed 3 times with PBS for 5 min. Next, the sections were stained with the strepavidin-biotin system followed by counterstaining with hematoxylin. Finally, all of the specimens were sealed with neutral glue.

### Cell cultures

PrC primary cell cultures were obtained from fresh samples of patients with prostate adenocarcinoma. The protocol for obtaining the sample and its use was approved by the Universidad de Chile Bioethics Committee including the required informed consent (DI MULT 05/36-2 project Universidad de Chile). The human prostate carcinoma cell line (PC3) was obtained from the American Type Culture Collection (Rockville, MD, USA). PC3 cells were cultured in Dulbecco’s modified Eagle’s medium (DMEM) supplemented with 10% fetal bovine serum and 1% antibiotic penicillin and streptomycin. PrC primary cell cultures were grown in DMEM supplemented with 7% fetal bovine serum and 1% antibiotic penicillin and streptomycin. All the cells were maintained under standard cell culture conditions at 37°C in 5% CO_2_ in a humid environment (32).

### Cell treatments

Anandamide (dissolved in DMSO), 2-arachidonoyl glycerol (2-AG; dissolved in DMSO) and methanandamide (Me; dissolved in methanol) were used for the treatment of cells. The final concentrations of DMSO and the methanol used were proven harmless for the treated cells. For the dose-dependent studies, the cells were treated with Ana, 2-AG and Me at final concentrations of 2.5, 5.0 and 10.0 μM for 48 h. For the rest of the experiments 5.0 μM was used since it showed the best concentration/effect ratio. To establish the role of CB1 and CB2 receptors in the endocannabinoid effects, cells were treated with 20 μM of CB1 antagonist SR141716 (diluted in DMSO) for 30 min at 37°C in the absence of light. Afterwards, the antagonist was removed and the cells were washed with PBS. Finally, the cells were treated with the different endocannabinoids under the same conditions as explained above.

### Cell viability

The effect of endocannabinoids on cell viability was determined by 3-(4,5-dimethylthiazol-2-yl)-2,5-diphenyl tetrazoliumbromide (MTT) assays. The cells were plated at 5×10^3^/well in 200 μl of complete culture medium containing 2.5, 5.0 and 10.0 μM concentrations of Ana, 2-AG or Me in 96-well microtiter plates for 48 h at 37°C in a humidified chamber. Each condition was repeated 5 times. After incubation, MTT reagent (100 μl 5 mg/ml in PBS) was added to each well, and the microplates were incubated for 3 h at 37°C in the dark. The MTT solution was removed from the wells by aspiration and the crystals were dissolved in DMSO (150 μl). Absorbance was recorded on a microplate reader (Mod. DNM-9602; Perlong, Beijing, China) at a 550 nm wavelength. The effect of the three endocannabinoids on growth inhibition was assessed as the percentage of inhibition in regards to the untreated controls (100%).

### Western blot analysis

Following the treatment of cells with endocannabinoids at a concentration of 5 μM for 48 h, the medium was aspirated and the cells were washed with PBS and then trypsinized and centrifuged at 2,500 rpm for 5 min. The resulting pellet was resuspended in a lysis buffer with a protease inhibitor cocktail. Later, the cells were scraped and the lysate was collected in a microfuge tube and passed through a syringe to break up the cell aggregates. The lysate was cleared by centrifugation at 13,500 × g for 15 min at 4°C, and the supernatant was discarded and the protein pellet was collected for protein quantification using the Bradford method at 570 nM in a Rayleigh spectrophotometer (UV-1600 model). For western blot analysis, 40 μg of protein was resolved over 10% polyacrylamide gels, with a molecular weight standard (cat. no. 161-0374; Pierce, Rockford, IL, USA), and electro-transferred onto a nitrocellulose membrane (cat. no. 162-0115; Bio-Rad, Berkeley, CA, USA). The nonspecific sites on the membranes were blocked by being incubated with a blocking buffer for 1 h at room temperature. Then, the membranes were incubated with the corresponding primary antibody in blocking buffer, overnight at 4°C, followed by incubation with anti-mouse or anti-rabbit peroxidase-conjugated secondary antibodies and detected by chemiluminescence (EZ-ECL kit, cat. no. 20-500-120; Biological Industries, Beit-Haemek, Israel). The bands were scanned and then analyzed using the scientific software program Un-Scan-It (Silk Scientific Corporation, Orem, UT, USA).

### Quantification of cell cycle distribution by flow cytometry

The cells were grown at a 10^6^ density in culture dishes and were treated with endocannabinoids at a 5.0 μM concentration for 48 h. Then, the cells were trypsinized, washed with PBS and fixed in cold ethanol (70% v/v) and stored in cytometry tubes at −20°C. At the time of the analysis, the cells were centrifuged and resuspended in a labeling solution (propidium iodide 0.5 mg/ml and RNase 100 μg/ml). The labeled cells were incubated for 30 min at 37°C in the dark and then analyzed in a FACScan cytometer (Becton-Dickinson, Franklin Lakes, NJ, USA). Data analysis was assessed using WinMD1 version 2.8 software.

### Annexin V assay

Cells were grown at a 10^6^ density and treated with endocannabinoids at a 5.0 μM concentration for 6 h. After the treatment, the cells were trypsinized, washed with cold PBS and then centrifuged at 2,500 rpm for 5 min. The supernatant was discarded and the pellet was resuspended in 1 ml of cold PBS. The cells were counted with a Neubauer chamber and 10^5^ cells were transferred to a cytometry tube. Then, the cells were processed and labeled according to the BD Pharmingen Annexin V-FITC Apoptosis Detection kit that was used for this assay. The labeled cells were analyzed in a FACScan cytometer (Becton-Dickinson). Data analysis was assessed using WinMD1 version 2.8 software.

### Statistical analysis

Data are expressed as mean ± SD. The significance between the control and treated cells was calculated using an unpaired t-test for P-value. P<0.05 was considered to indicate a statistically significant difference. The GraphPad Quick Calcs program was used.

## Results

### Immunohistochemistry of CB1 and CB2 receptors in prostatic tissue

**Figure 1:**
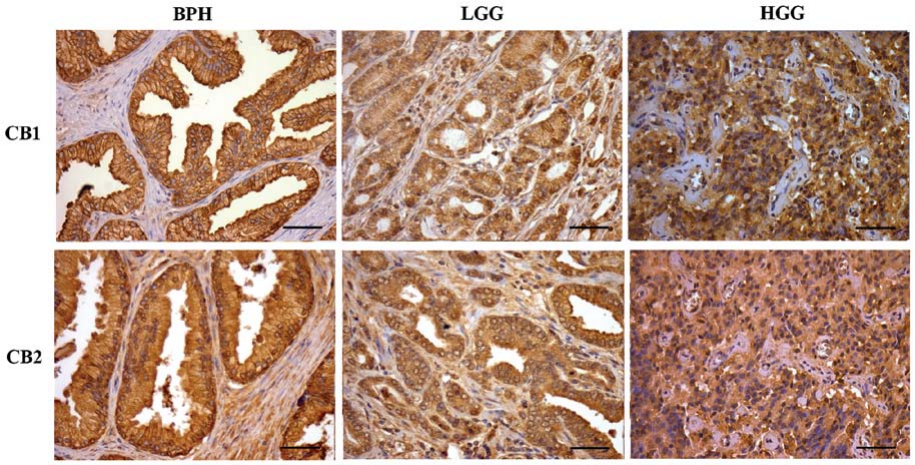
Immunohistochemical staining for CB1-R and CB2-R in BPH, LGG and HGG (x40 magnification). Scale bar, 50 μm. BPH, benign prostatic hyperplasia tissue; LGG, low Gleason grade prostatic cancer tissue; HGG, high Gleason grade prostatic cancer tissue; CB1-R, cannabinoid receptor 1; CB2-R, cannabinoid receptor 2.

The immunodetection of CB1 and CB2 receptors in PrC and BPH tissues is shown in Fig. 1. For both receptors, the staining pattern was similar. In BPH, the staining was strong in the apical cell membrane and mild in the cytoplasm. Low Gleason grade (LGG) and high Gleason grade (HGG) samples showed a similar pattern with homogeneous and diffuse cytoplasmic staining, but the intensity of the staining was stronger in the HGG samples. Exceptionally, there were positive nuclei of isolated lymphocytes.

Additionally, the presence of CB1 and CB2 receptors, in the PC3 cells and BPH and PrC primary cultures, was evaluated by immunocytochemistry and western blot analysis. Both types of cells showed the presence of the receptors. In addition, the receptors appeared to be homogeneously distributed throughout the cell surface of PC3, BPH and PrC cells in a similar way (data not shown).

### Effect of endocannabinoids on the cell viability of PC3 cells and primary cultures of BPH and PrC

**Figure 2:**
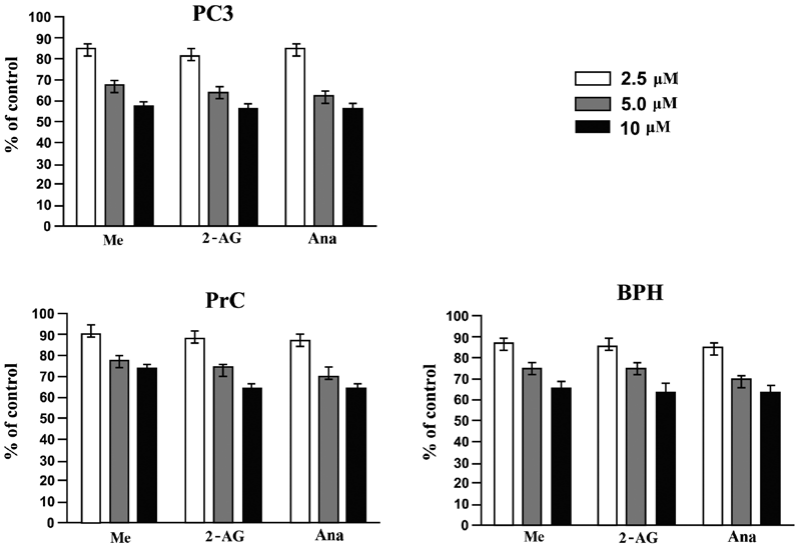
Effect of endocannabinoids on the viability of PC3 cells and primary cultures of BPH and PrC. PC3 cells and cultures of BPH and PrC were treated with different doses of endocannabinoids (2.5, 5.0 and 10.0 μM) for 48 h at 37°C. After the treatments, cell viability was evaluated through MTT assay (n=4). The results are expressed comparing the results to the viability of the control cells (cells that were left untreated) (P<0.05). BPH, benign prostatic hyperplasia; PrC, prostate cancer; Me, methanandamide; 2-AG, 2-arachidonoyl glycerol; Ana, anandamide.

To assess the cell viability response of the PC3 cell line and the primary cultures of PrC and BPH and the primary cultures to the different endocannabinoids, MTT assay was employed. Fig. 2 shows that treatment of PC3 cells and primary cultures of PrC and BPH for 48 h with the different endocannabinoids (at 2.5, 5.0 and 10 μM concentrations) significantly decreased the viability of the cells (P<0.05). Even more, the effect was proven to be dose-dependent and PC3 cells were more sensitive to the treatments than both primary cultures. Among the primary cultures, BPH cultures proved to be more sensitive to the treatments than the PrC cultures.

### Participation of CB1 and CB2 receptors in the effect by endocannabinoids on the viability of PC3 cells and primary cultures of BPH and PrC

**Figure 3:**
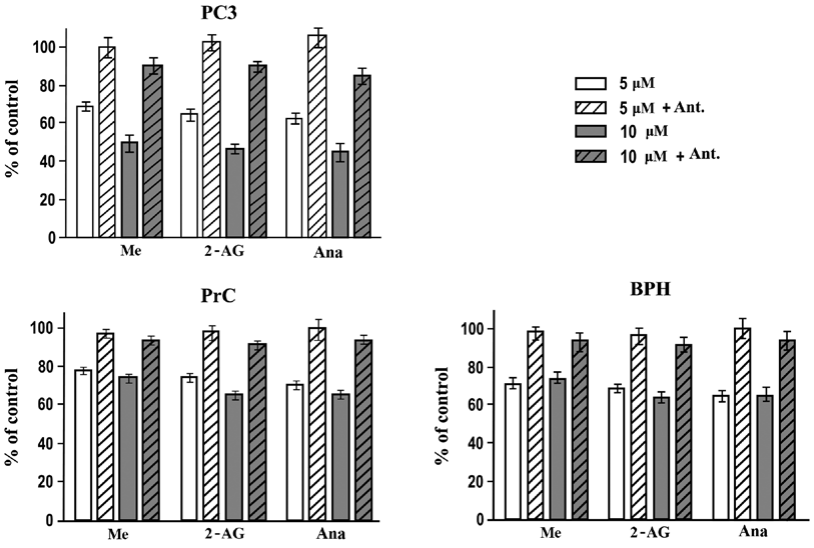
Role of CB receptors in the effect by endocannabinoids on the viability of PC3 cells and BPH and PrC cultures. The effect of a CB1 antagonist (Ant.) was studied through MTT assay to analyze the possible preventive effect of such an antagonist. PC3 cells, BPH and PrC cultures were incubated with SR141716 (a CB1 antagonist) for 30 min at 37°C. After incubation with the antagonist, the cells were treated as explained in fig. 2 legend but with just two different concentrations of endocannabinoids (5.0 and 10.0 μM). The effects on cell viability were evaluated through MTT assay and are expressed comparing them with the untreated cells (n=3). BPH, benign prostatic hyperplasia tissue; PrC, prostate cancer; CB1-R, cannabinoid receptor 1; Me, methanandamide; 2-AG, 2-arachidonoyl glycerol; Ana, anandamide.

To study the role of CB1 and CB2 receptors in the endocannabinoid-mediated suppression of viability of the different cell cultures, we evaluated the effect of a CB1 antagonist SR141716 using the MTT assay. Cells were incubated with 20 μM SR141716 for 30 min (which had no effect on the cell viability) and then were treated normally with the different endocannabinoids (Fig. 3). The pretreatment with the antagonist showed a significant protective effect on both PC3 cells and primary cultures of BPH and PrC. Only by using a 10 μM concentration of the endocannabinoids were we able to observe a small effect on the cell viability again (P<0.05) but only in the PC3 cells. The effect of the treatments on both primary cultures remained the same even at the highest dose used. These results showed that the activation of the CB1 receptors was required in the endocannabinoid-mediated growth inhibition process or cell death noted in PC3 cells and primary cultures of BPH and PrC when treated with endocannabinoids.

### Effect of endocannabinoids on the cell cycle distribution of PC3 cells and primary cultures of BPH and PrC

**Figure 4:**
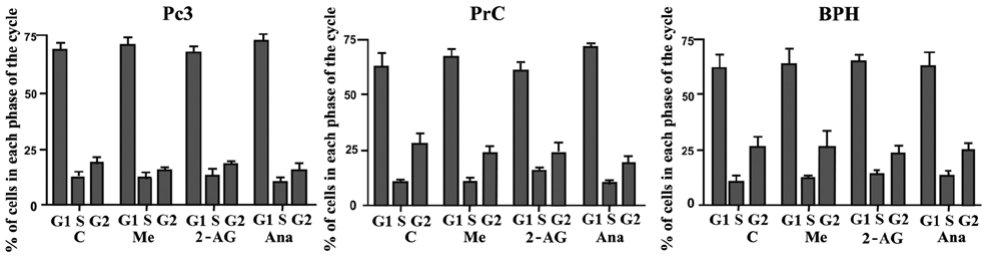
Effect of endocannabinoids on the cell cycle distribution of PC3 cells and BPH and PrC cultures. Cell cycle analysis was assessed to determine whether the effect on cell viability noted in the MTT assays could be cell cycle-dependent. PC3 cells and primary cultures of BPH and PrC were treated and then labeled. The labeled cells were analyzed using a FACScan cytometer, and the percentage of cells in the G1, S and G2 phases were calculated using the WinMDI v2.8 software (n=3, P>0.05). BPH, benign prostatic hyperplasia tissue; PrC, prostate cancer; C, control; Me, methanandamide; 2-AG, 2-arachidonoyl glycerol; Ana, anandamide.

Previous research from our group showed a decrease in the expression of PCNA (a cell proliferation marker) in response to endocannabinoid treatment (data not shown). Due to these results, we hypothesized that the effect on cell viability demonstrated by the endocannabinoids on the different cell cultures could be produced through induction of apoptosis by cell cycle arrest. PC3 cells and primary cultures of BPH and PrC were treated with the different endocannabinoids for 48 h at a 5 μM concentration. As shown in Fig. 4 there were no significant variations in the distribution of cells throughout the cell cycle when comparing control untreated cells with PC3 cells and PrC and BPH cultures that were treated with the different endocannabinoids (P>0.05).

### Flow cytometric analysis of FITC Annexin V staining in the PC3 cells and primary cultures of PrC

**Figure 5:**
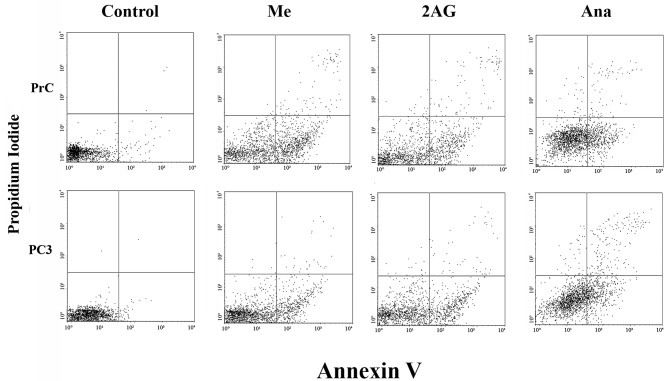
Flow cytometric analysis of FITC Annexin V staining. Primary cell cultures and cell lines (PC3) of prostate cancer were left untreated (Control; first panels at the left) or treated for 6 h with endocannabinoids at a concentration of 5 μM (second, third and fourth panels from the left). Cells were incubated with FITC Annexin V in a buffer containing propidium iodide (PI) and then analyzed by flow cytometry. Untreated cells were mostly FITC- and PI-negative which indicates that they were not undergoing any type of cell death process. After the 6-h treatment there were two different groups of cells: one composed of viable cells (FITC- and PI-negative, lower left quadrant) and one composed of cells undergoing apoptosis (FITC-positive and PI-negative, lower right quadrant). Another very small population of cells was observed to be both FITC- and PI-positive (upper right quadrant) which indicates that they were in the final stage of apoptosis or undergoing necrosis (n=3). Me, methanandamide; 2-AG, 2-arachidonoyl glycerol; Ana, anandamide.

To analyze the action of endocannabinoids on the different cultures of PrC cells and the possibility of a proapoptotic effect we used a FITC Annexin V assay. As shown in Fig. 5, the number of cells in the apoptotic state was higher in all of the treated cell cultures when compared with the untreated ones, while the number of necrotic cells remained statistically without variation (P>0.05). This effect was noted in both types of cells, but it was greater in the PC3 cells than that noted in the primary cultures of PrC. In addition, none of the different endocannabinoids showed a higher effect when compared with the others in the cell lines, yet the results varied among the different endocannabinoids in the primary cultures, with Me showing a higher effect than the other two agents. These results suggest that the effect of endocannabinoids on PC3 cells and primary cultures of PrC may be caused by the activation of the apoptotic pathway.

### Effect of endocannabinoids on the expression of active caspase-3 and Bcl-2 in primary cultures of PrC and BPH

**Figure 6:**
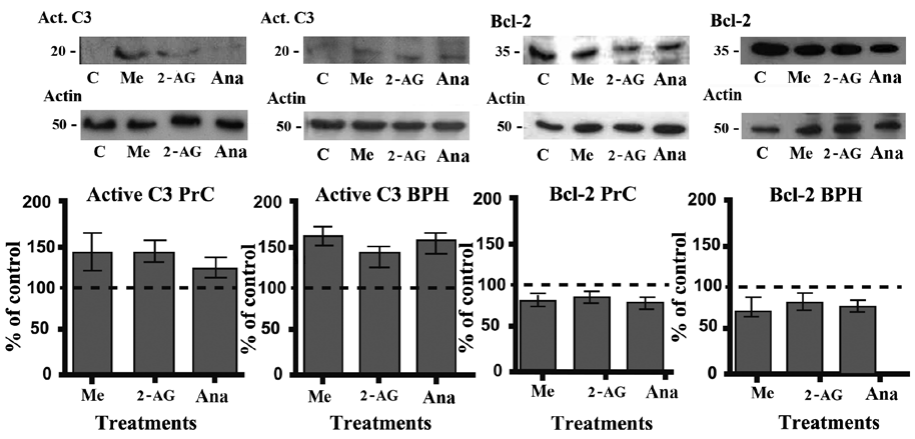
Expression of active caspase-3 and Bcl-2 in primary cultures of PrC and BPH after treatment with endocannabinoids. Primary cultures of PrC and BPH were treated with endocannabinoids at a 5 μM concentration for 48 h. After the treatments, the cells were collected and proteins were extracted and analyzed through western blot analysis. The figures show the variation in active caspase-3 and Bcl-2 expression in the treated cells compared to the expression in the control untreated cells. The dotted lines represent the control untreated cells (100%). Each image shows a representative experiment repeated three times with similar results (n=3) (P<0.05 in all cases). BPH, benign prostatic hyperplasia; PrC, prostate cancer; C, control; Me, methanandamide; 2-AG, 2-arachidonoyl glycerol; Ana, anandamide.

The results of the Annexin V assay indicated that endocannabinoids may exert their action through the activation of the apoptotic pathway. To corroborate this we analyzed their effect on the expression of important proteins of the classical apoptotic pathway such as the active form of caspase-3 and Bcl-2. Cells were treated for 48 h with the different endocannabinoids at a 5 μM concentration. After the treatments (Fig. 6), there was a significant increase in the level of active caspase-3 and a consequent decrease in the level of Bcl-2 (both in PrC and BPH cultures), which corresponds with an activation of the classical apoptotic pathway.

### Effect of endocannabinoids on the expression of Erk and Akt in primary cultures

**Figure 7:**
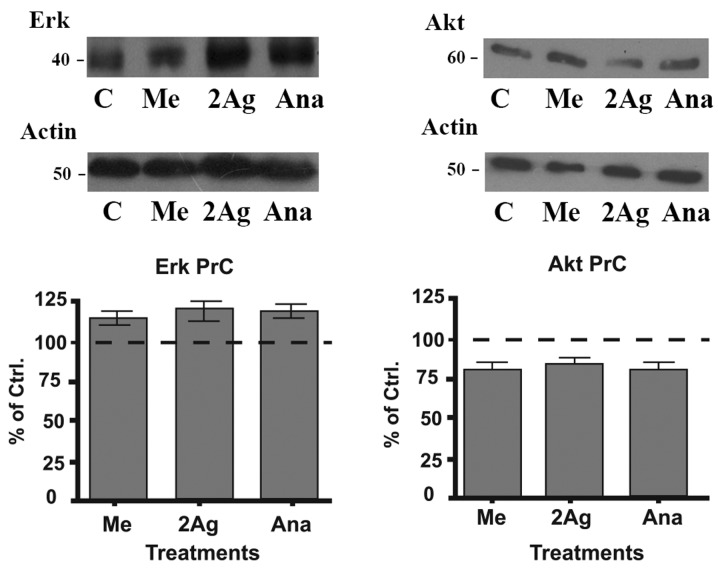
Effect of endocannabinoids on the expression of Erk and Akt in primary cultures of PrC. As detailed in Materials and methods, primary cultures of PrC were treated with endocannabinoids at a 5 μM concentration for 48 h. After the treatments, the cells were collected and proteins were extracted and analyzed through western blot analysis. The figures show the variation of Erk and Akt expression in the treated cells compared to these levels in the control untreated cells. Each image shows a representative experiment repeated three times with similar results (n=3) (P<0.05). PrC, prostate cancer; C, control; Me, methanandamide; 2-AG, 2-arachidonoyl glycerol; Ana, anandamide.

It has been shown that the Erk and Akt signaling pathways play a very important role in regulating the cellular response to proliferative signals and that mutations in these pathways are often observed in various types of cancer (26). Therefore, we analyzed the effect of endocannabinoids on the expression of these proteins to test the possibility of their involvement in the apoptotic process as noted in the above experiments. Cells were treated with different endocannabinoids for 48 h at a 5 μM concentration. After the treatments, we observed that Erk protein expression was augmented in the treated cells when compared to the control-untreated cells (Fig. 7). In contrast, the levels of Akt decreased following all the treatments in contrast to the increase noted in the Erk expression. These results indicate that Erk activation/Akt downregulation may be involved in the suppresive effect that endocannabinoids have on the cell viability of PrC cells.

## Discussion

Prostate cancer has become the most commonly diagnosed cancer in men and is one of the most threatening diseases in Western countries. Although there are therapies that have been proven effective, such as androgen deprivation, these treatment strategies are not able to eliminate all tumor cells. In addition, current chemotherapy treatments cause many undesirable side effects for patients.

It has been reported that endocannabinoids have a wide range of regulatory effects in a variety of physiological processes. One of their most promising actions has to do with their effect on various types of cancers and their potential use in the treatment of these diseases (18).

In the present study, it was demonstrated by immunohistochemistry that CB1 and CB2 receptors are highly expressed in prostate cancer samples with different degrees of malignancy, as well as in BPH tissue. The presence of receptors CB1/CB2 in commercial cell lines (PC3), as well as in primary cultures of PrC, was additionally demonstrated by immunocytochemistry and western blot analysis (data not shown) as it was coincidentally reported by other studies (25,30,33). Furthermore, we observed that the expression of these receptors was associated with the degree of malignancy in PrC, with higher expression in the most aggressive samples of PrC. To analyze the effect that the analogs of endocannabinoids have on the various PrC cell cultures, the sensitivity to three different concentrations of endocannabinoids was evaluated, which established that primary cultures were more resistant to endocannabinoids than PC3 cells. This differential effect may be explained by a different expression of metabolizing enzymes, such as FAAH, which metabolize and degrade endocannabinoids *in situ*. Several studies have illustrated differences in the expression of these enzymes in prostate cancer cell lines and the effect this has on treatment with endocannabinoids in cancer cells (34,35). The action of these enzymes, their expression and related metabolism of endocannabinoids may account for the weaker effect that the treatments had on the primary cultures. Moreover, it was also observed that the effect of the endocannabinoids in regards to the viability of the different cell cultures occurred in a dose-dependent manner and that there may be a saturation of the receptors associated with the increasing concentration of each treatment.

Furthermore, the effect of the different treatments on both cell lines and primary cultures was almost totally reversed when they were previously incubated with the CB1R antagonist SR141716. As this is a selective antagonist for the CB1 receptor, the results obtained lead us to conclude that the observed effect of the different treatments was produced mainly by the activation of CB1 and not CB2; these results also discard a possible receptor-independent effect of the endocannabinoids.

Olea-Herrero *et al* (36) recently postulated that the effect of methanandamide on the survival of PC3 cells may be carried out specifically by activation of the CB2 receptor. This finding not only differs from our results but also of the published data by Agudelo *et al* (37); who despite not using methanandamide used its endogenous analog anandamide. This group reported that the effect of anandamide was produced by the activation of CB1 receptors. Furthermore, the affinity of methanandamide for the CB2 receptor was very low (with a Ki of 815 nM for CB2 and a Ki of 20 nM for CB1). Some researchers even consider this molecule to be a specific agonist for CB1. In previous experiments from our laboratory using radioligand binding studies, we observed that in both cell lines and primary cultures, endocannabinoids bound more readily to CB1 receptors than to CB2 receptors (data not shown). This may account for the effect observed when using the specific inhibitor for CB1.

Although previous results depicted a decrease in PCNA in PrC cell lines following endocannabinoid treatment, the cell cycle analysis assessed by flow cytometry showed no variations in the distribution of the different stages of the cell cycle. Other studies have reported cell cycle arrest following treatment with different THC analogs but using them at higher concentrations (39,40), which may be one reason why even though we previously observed that levels of PCNA were lower after the treatments, that was not enough to modify the cell cycle distribution of the different cell cultures. Another reason for this may be that the decrease in the PCNA levels was not high enough to have a significant effect on the cell cycle (38).

Once we concluded that the effect of endocannabinoids on cell viability was not exerted through a modification of the cell cycle, we analyzed whether this effect may be due to activation of the apoptotic pathway. Through Annexin V assays we observed no change in the percentage of necrotic cells after the treatment with endocannabinoids, but we observed an increase in the number of cells in early apoptosis after the treatments. These results were confirmed by evaluating the expression of caspase-3 and Bcl-2 post-treatment with the endocannabinoids. The results revealed an increase in the levels of active caspase-3 and a decrease in the expression levels of Bcl-2. This simultaneous effect observed in both proteins may be explained by a change in the activity of the nuclear factor-κB (NF-κB) induced by the endocannabinoids. NF-κB is involved in cell proliferation through multiple mechanisms, some of which control the expression of Bcl-2 and also the activation of proteins involved in the apoptotic pathway such as caspases. It has been reported that in prostate cancer NF-κB is overactivated, thus endocannabinoids may act through this pathway, simultaneously activating caspase-3, downregulating Bcl-2 expression; and other proteins that may also be involved in the apoptotic process (7,41).

All of our results suggest that endocannabinoids may exert their effect by activating the apoptotic pathway without modifying the cell cycle stage or inducing necrosis. Moreover, a decrease in the levels of AKT in the primary cultures was detected after treatment with endocannabinoids. AKT functions as a critical positive regulator of metabolism and cell proliferation (39) which is positively correlated with the effect caused by the different treatments on prostate cancer cultures. These results are supported by other reports that have shown that AKT is decreased in other types of cancer following treatment with THC (41). Furthermore, increases in the activation of Erk and treatment with cannabinoids have been correlated with antiproliferative processes in other types of cancer (42). The results observed in our study depict a simultaneous activation of the ERK pathway and a decrease in AKT levels. The combination of these two events may contribute to the activation of antiproliferative pathways and a decrease in the proliferative pathways in primary cultures of PrC. A more in-depth analysis of the proteins involved in these pathways may be important to better describe the intracellular mechanisms that may be activated by endocannabinoids in prostate cancer cells and thus search for other possible targets of intervention to potentiate the effects depicted by the treatment of cancer cells with endocannabinoids.

Finally and in accordance with our findings, we conclude that endocannabinoids are capable of halting the growth of prostate cancer cells through activation of apoptotic mechanisms. Furthermore, we suggest that this effect may be through the modulation of the Erk and Akt signaling pathways by endocannabinoids. Therefore, endocannabinoids appear to be a powerful tool for investigation in the development of drugs and treatments against advanced PrC.
